# “Non-Triangle Plane” Surgical Technique of Video-Assisted Thoracic Surgery Atypical Segmentectomy for Stage IA Non-Small-Cell Lung Cancer: Early Experience

**DOI:** 10.3389/fsurg.2021.731283

**Published:** 2021-10-26

**Authors:** Chao Zhou, Jun Qian, Wentao Li

**Affiliations:** ^1^Department of Thoracic Surgery, Shanghai Chest Hospital, Shanghai Jiao Tong University, Shanghai, China; ^2^Department of Cardiothoracic Surgery, Dehong People's Hospital, Affiliated Dehong Hospital of Kunming Medical University, Dehong, China

**Keywords:** anterior segmentectomy, complex segmentectomy, intersegmental plane, non-small-cell lung cancer, pulmonary function

## Abstract

**Objectives:** To evaluate the safety and feasibility of a novel surgical technique (“non-triangle plane” technique) of two-port (mini-utility) video-assisted thoracic surgery (VATS) atypical segmentectomy (S^3^+S^1+2^c) with tunneling stapler for small-sized non-small-cell lung cancers (NSCLCs) located in left S^3^ close to the intersegmental plane between S^3^ and S^1+2^c.

**Materials and Methods:** This retrospective descriptive study included 16 patients who, between April 2016 and December 2019, underwent a single two-port (mini-utility) VATS atypical segmentectomy (S^3^+S^1+2^c) with tunneling stapler technique for small-sized NSCLCs with a ground-glass opacity (GGO) rate of more than 50% by a constant surgical team in two hospitals. Perioperative data and survival data were collected and retrospectively analyzed. Postoperative follow-up was performed every 6 months.

**Results:** Six patients were with adenocarcinoma *in situ*, and ten were with minimally invasive adenocarcinoma. The mean surgical margin was 14.06 ± 3.02 mm. The mean operation time was 53.88 ± 9.76 min. The mean duration of chest tube drainage was 4 ± 1.21 days, and the median length of postoperative hospital stay was 4 days. There was no perioperative morbidity and mortality. The median follow-up was 47.5 months (17–61 months). No recurrences occurred, and all patients were still alive at the last registered follow-up (May 31, 2021).

**Conclusion:** Two-port (mini-utility) VATS atypical segmentectomy (S^3^+S^1+2^c) with tunneling stapler technique is a safe and feasible option for the treatment of small-sized NSCLCs located in left S^3^ close to the intersegmental plane between S^3^ and S^1+2^c.

## Introduction

Lobectomy with mediastinal lymph node dissection is a standard surgical treatment for early-stage non-small-cell lung cancer (NSCLC) (1). With the new application of low-dose computed tomography (LDCT), early diagnosis of ground-glass nodules (GGNs) and early-stage NSCLCs has sharply increased (2). Most of GGNs that are pure ground-glass opacity (GGO)-dominant are adenocarcinoma *in situ* (AIS) or minimally invasive adenocarcinoma (MIA) with good pathological prognoses (3–5). Limited resection and minimally invasive surgery, especially segmentectomy, are preferred for stage IA NSCLC uncompromised patients (6–8).

**Figure 1:**
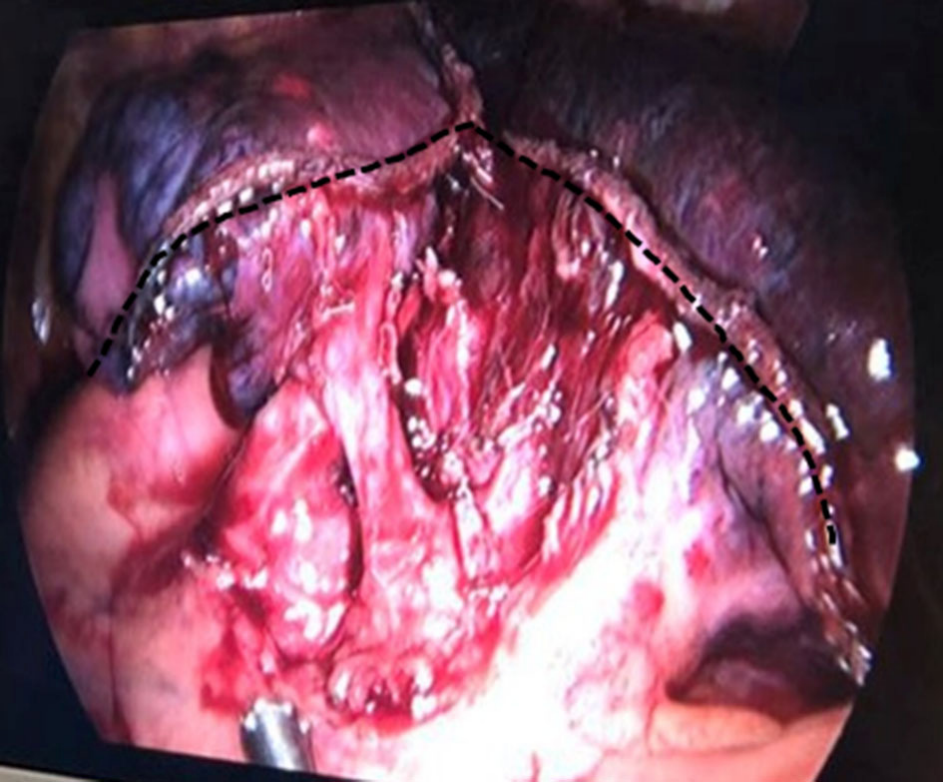
Dotted line demonstrates that after left anterior segmentectomy, most conventional segmental planes are like a triangle.

Currently, segmentectomy is a popular surgical treatment for early-stage NSCLC. However, the application of complex segmentectomy, which can create more than one or intricate intersegmental planes, is still controversial (9). This type of surgery is associated with an increased risk of complications (prolonged air leakage, bleeding, and so on) and local recurrences (insufficient surgical margin) compared to simple segmentectomy. Left anterior segmentectomy is a complex segmentectomy, in which an intersegmental plane is a triangle plane (Figure 1). The conventional segmental plane dissection might result in short surgical margins (not tumor-free), especially for the tumor located close to intersegmental planes, and restriction of residual lung re-expansion (postoperative atelectasis). It is usually incorporated within the resection of the upper three segments of the left upper lobe. Therefore, it is possible that it could lead to higher hospitalization costs and reduced pulmonary function for patients with small-sized NSCLC.

In this study, we evaluated the safety and feasibility of a novel surgical technique, also known as “non-triangle plane” technique, of two-port (mini-utility) VATS atypical segmentectomy (S^3^+S^1+2^c)) with tunneling stapler for small-sized NSCLCs located in left S^3^ close to the intersegmental plane between S^3^ and S^1+2^c, and we retrospectively analyzed our clinical data.

## Materials and Methods

This study was approved by the Ethics Committee of Shanghai Chest Hospital and Dehong People's Hospital, and individual written informed consent was obtained from all patients.

### Patient Selection

This retrospective descriptive study included 16 patients who underwent single two-port (mini-utility) VATS atypical segmentectomy (S^3^+S^1+2^c) with tunneling stapler technique for small-sized NSCLCs with a GGO rate of more than 50%, from April 2016 to December 2019. Surgery was performed by a surgical team of the thoracic surgery department of Shanghai Chest Hospital. All patients were followed up at least 1 year before surgery.

All patients received a thorough preoperative evaluation in our hospital, including history, physical examination, serum biochemical analysis, thoracic CT, abdominal ultrasound scan, MRI of the brain, and pulmonary function tests. Bone scanning was carried out in selected patients. All GGNs were located in the left S^3^ close to the intersegmental plane between S^3^ and S^1+2^c. All the lesions were considered to be cT_1_N_0_M_0_ NSCLCs.

General anesthesia with single-lung ventilation using a double-lumen endotracheal tube was utilized in all patients. All patients were moved to the ward after operation, provided they were hemodynamically stable. Special attention was given to early postoperative mobilization, effective pulmonary function exercise, and effective coughing. The chest tube was removed in the absence of air leakage, 24-h drainage of <200 ml, and a normal chest radiograph. The patient was discharged the same day.

Postoperative follow-up was performed every 6 months. Evaluation modalities for the follow-up included tumor markers, CT, and abdominal ultrasound scan. Follow-up pulmonary function was essential 6 months after surgery. Recent patient status was ascertained by using the medical records of outpatient clinics or by telephone interview.

### Pulmonary Function Tests

Forced vital capacity (FVC) and forced expiratory volume in 1 second (FEV1) were measured by spirometry within one wk before surgery and 6 months after surgery, respectively. The loss in pulmonary function variables at 6 months after surgery was calculated by the formula proposed by Fang et al. (10): *FVC loss* = *(preoperative FVC-postoperative FVC)/preoperative FVC*×*100%*. Average FVC loss per segment = FVC loss/1.3 S^3^:1+S^1+2^c:1/3 = 1.3).

### Surgical Technique

#### Patient Positioning

The patient was placed in the right lateral decubitus position. A pillow was placed under the chest of the patient to get more widened intercostal spaces. The surgeon and assistant stood in front of the patient.

#### Approach

The approach consisted of two mini ports with the size of skin incisions of 0.8–1 cm. The observation port was placed at the seventh intercostal space (ICS) on the middle axillary line, after which a thoracoscope was inserted at 30°. The working (utility) port was placed at the fourth ICS on the anterior axillary line.

#### Surgical Procedure

First, the oblique fissure and posterior mediastinal pleura were opened. Interlobar lymph nodes (#11) were removed and confirmed negative for metastasis by intraoperative frozen-section pathology. The apicodorsal artery (A^1+2^a+b, A^1+2^c) and lingular division artery (A^4+5^) were then exposed. The horizontal branch of the apicodorsal artery (A^1+2^c) was divided by a stapler.

**Figure 2:**
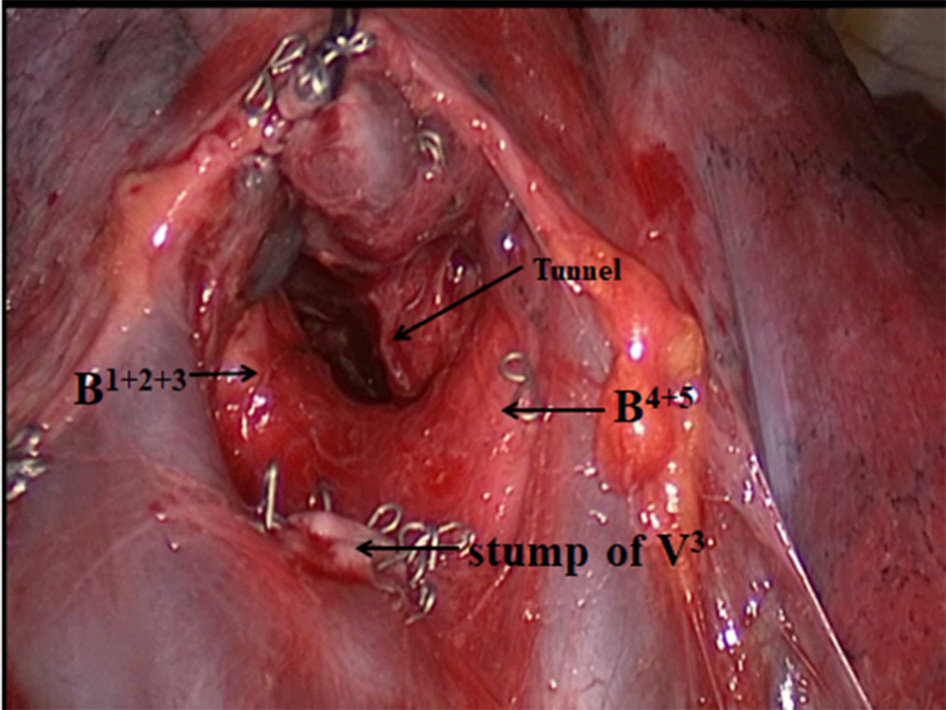
Surgical view immediately before intersegmental tunneling. Arrows represent the tunneling point.

Second, the upper lobe was gently retracted backward. The pleura covering the superior pulmonary vein's surface was then opened, and the anterior vein (V^3^) was dissected. The V^3^ was divided by a stapler. Intersegment lymph nodes (#13) were removed and confirmed to be negative for metastasis by intraoperative frozen-section pathology. Instruments, including long kelly, forceps, or suction tube, were then used to bluntly dissect along the medial sidewall of lingular division bronchus (B^4+5^) toward the back at the fissure. Intersegmental tunneling was performed along the root of the segmental bronchus between the upper-division bronchus (B^1+2+3^) and B^4+5^ (Figure 2). The anterior bronchus (B^3^) was then dissected and divided by a stapler. We used the suction tube to bluntly dissect and enlarge the space again. If the resected margins could be ensured, there was no need to divide the anterior artery (A^3^). Segmental arteries and bronchi are good anatomical landmarks for intersegmental planes. The segmental plane between S^1+2^a+b and S^3^+S^1+2^c was divided along the apicodorsal vein (V^1+2^a-c) and A^3^ by staplers. The segmental plane between S^3^+S^1+2^c and S^4+5^ was divided along the lingular vein (V^4+5^) and B^4+5^ by staplers. The important point was not to damage V^1+2^a-c, A^3^, and V^4+ 5^.

Finally, direct sutures of segmental planes with 7# silks between S^1+2^a + b and S^4+5^ were performed to avoid torsion of completely divided segments and decrease air leakage. Intraoperative frozen sections revealed adenocarcinoma. Lymph node sampling was subsequently completed. A 24F chest tube was then placed, and the wound was closed.

### Statistical Analyses

Data were collected and analyzed using SPSS for windows version 23.0 (SPSS, Inc., Chicago, United States). Continuous variables with normal distribution were expressed as mean ± standard deviation (x ± SD). Continuous variables with skewed distribution were described using the median. Categorical variables were described with number and percentage.

## Results

Patient characteristics and surgical outcomes are shown in Table 1. From April 2016 to December 2019, 16 patients (7 males and 9 females), with a mean age of 61.94 ± 11.39 yr (range, 37–80 yr), underwent a single utility Miniport VATS atypical segmentectomy (S^3^+S^1+2^c). All patients were asymptomatic. The mean tumor size was 9.5 ± 1.46 mm. The FVC loss and FEV1 loss were −12.78 and −13.81%, respectively. Average FVC loss per segment and average FEV1 loss per segment were −9.8 and −10.6%, respectively. Mean operating time and median blood loss were 53.88 ± 9.76 min and 90 ml, respectively. Mean postoperative day 1 drainage was 160.94 ± 58.18 ml. The mean chest drain duration was 4 ± 1.21 days (range, 2–7 days). The median length of postoperative hospital stay was 4 days. Histological diagnoses of six patients were AIS, and the others were MIA. The distribution of the stages, calculated using the eighth edition of the American Joint Committee on Cancer TNM classification system (10–13), is shown in Table 1. There was no perioperative morbidity and mortality. The median follow-up was 47.5 months (range, 17–61 months). No recurrences occurred, and all patients were still alive at the last registered follow-up (May 31, 2021).

**Table 1:** Patient clinicopathologic characteristics.

**Characteristic**	**Results**
**Age (years), mean±SD**	61.94 ± 11.39
**Gender**, ***n*** **(%)**	
Male	7 (43.8%)
Female	9 (56.2%)
**Smoking history**, ***n*** **(%)**	
Yes	5 (31.2%)
No	11 (68.8%)
**Pulmonary function, mean** **±** **SD**	
PreFVC(L)	2.66 ± 0.47
Pre FEV1(L)	2.1 ± 0.41
Post FVC(L)	2.32 ± 0.46
Post FEV1(L)	1.81 ± 0.35
FVC loss (%)	12.78%
FEV1 loss (%)	13.81%
**Mean distance to pleura (mm), mean** **±** **SD**	17.13 ± 2.39
**Tumor size (mm), mean** **±** **SD**	9.5 ± 1.46
**Postoperative outcomes**	
Operating time (min), mean±SD	53.88 ± 9.76
Median blood loss (ml)	90
Chest drain duration (days), mean ± SD	4 ± 1.21
POD 1 drainage (ml), mean ± SD	160.94 ± 58.18
Median LPOS, (days)	4
Morbidity, n	0
Mortality, n	0
**Surgical margin (mm), mean** **±** **SD**	14.06 ± 3.02
**Histological type**, ***n*** **(%)**	
Adenocarcinoma in situ	6 (37.5%)
Minimally invasive adenocarcinoma	10 (62.5%)
**TNM classification**, ***n*** **(%)**	
TisN0M0, 0	6 (37.5%)
T1a(mi)N0M0, IA	10 (62.5%)

*Pre, preoperative; Post, postoperative; FVC, forced vital capacity; FEV1, forced expiratory volume in 1s; LPOS, length of postoperative hospital stay; POD, postoperative day; SD, standard deviation*.

## Discussion

With the development of surgical techniques, VATS segmentectomy has been identified as an effective alternative approach in the treatment of small-sized NSCLC. In a propensity-matched study, VATS segmentectomy showed equivalent oncological outcomes compared to VATS lobectomy for early-stage NSCLCs (8). Left anterior segmentectomy is defined as a complex segmentectomy because of more than one intricate intersegmental plane and a more complicated procedure. However, there are still some controversies regarding complex segmentectomy (9). Main concerns include the failure to control cancer, i.e., insufficient surgical margin. In this study, mean distance to pleura of the tumors was 17.13 ± 2.39 mm. Wedge resection may result in bleeding because of thicker lung tissue for the deep tumors, and not perform a regional lymph node assessment. For the tumor located on the border of the left S^3^, VATS anterior segmentectomy may have a higher risk of local recurrences than multi-segmentectomy or additional subsegmentectomy. Consequently, most thoracic surgeons undergo tri-segmentectomy instead of anterior segmentectomy. Nonetheless, it is logical to assume that the selection of surgical type is determined by tumor location. We presented the new concept about atypical segmentectomy, which centering on the lesion to obtain adequate surgical margins. The median surgical margin was 13 mm in this study. We suggested if macroscopic surgical margin was 10 mm or more, it was adequate for patients with AAH, AIS, and MIA. Suzuki et al. reported that a surgical margin 5 mm or more could be considered to be adequate for non-invasive lung adenocarcinoma 2.0 cm or less in size (14).

Preoperative three-dimensional computed tomography bronchography and angiography can help to locate pulmonary nodules (15). In this study, clock dial-integrated positioning for small pulmonary nodules was applied in VATS. Li et al. (16, 17) reported the new method of intraoperative non-invasive localization and the accuracy of clock dial-integrated positioning for small pulmonary nodules is up to 99.0% (313/316). We believe that combined with the clinical treatment experience, the clock dial-integrated positioning method is effective for positioning in VATS. Meanwhile, our results showed that a novel atypical segmentectomy could manage the tumor located in the left S^3^ close to the intersegmental plane between S^3^ and S^1+2^c under the condition of preserving pulmonary function and obtaining adequate surgical margins (Supplementary Material Video 1) with short- and long-term acceptable outcomes.

**Figure 3:**
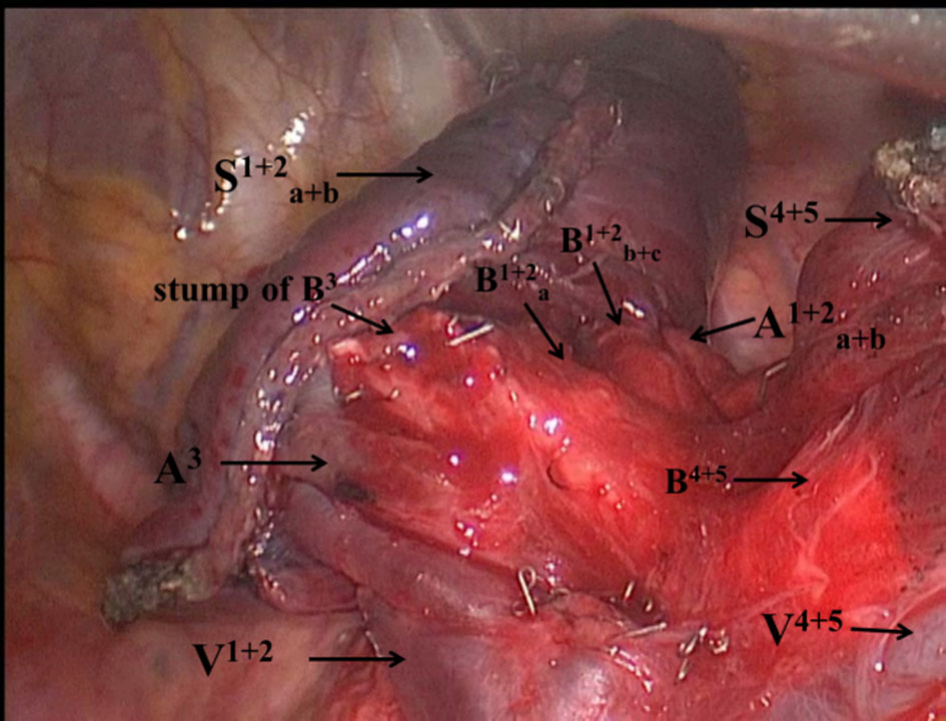
The remaining left upper lobe was divided into two separate segments (S${}_{\text{a}+\text{b}}^{1+2}$ and S^4+5^).

The intersegmental plane of the left anterior segmentectomy is a triangle plane. The re-expansion of the residual segments in the remaining lobe could be restricted because of stapler division and angled intersegmental plane, thus resulting in decreased preserved pulmonary function (18, 19). However, Tao *et al* reported that using a stapler does not result in less-preserved pulmonary function compared to electrocautery in the division of the intersegmental planes (20). Furthermore, Fang *et al*. suggested that the average loss of spirometry indexes after VATS segmentectomy with stapler division was significantly greater than after VATS lobectomy by comparing the average loss of pulmonary function per segment resected between lobectomy and segmentectomy groups (FVC loss per segment: −5.3 vs. −10.4%, *p* < 0.001; FEV1 loss per segment: −5.8 vs. −12.7%, *p* < 0.001) (21). However, our results of the average loss of pulmonary function per segment resected were slightly lower than that reported by Fang *et al* (FVC loss per segment: −9.8 vs. −10.4%; FEV1 loss per segment: −10.6 vs −12.7%). We believe that the linear intersegmental plane had less effect on postoperative pulmonary function. Preserved pulmonary segments (S^1+2^a+b and S^4+5^) in the upper lobe were completely separated and fully re-expanded (Figure 3). Two linear intersegmental planes were sutured to avoid torsion of segments and prolonged air leakage. Handa *et al*. reported that the linear intersegmental plane could decrease prolonged air leakage more effectively compared to several intricate intersegmental planes (22). “Non-triangle plane” tunneling staple technique can avoid restricting re-expansion of the residual segments in the remaining lobe and reduce the incidence of air leakage and bleeding. In this study, no prolonged air leakage occurred.

It is worth mentioning that our approach is two-port (mini-utility). The skin incision is smaller compared to other approaches; the muscle and pleural trauma is also smaller, there is less postoperative pain, and the cosmetic effect is better. Because of two-miniport, the direction of stapler insertion is less limited than uniport. In addition, the functions of the observation port and the working (utility) port are interchangeable according to surgical requirements, while there is less trauma than in the three-port.

### Limitation of this Study

This was a retrospective study, and the data were obtained from a constant surgical team's patients. Number of patients is very small but of course is a very infrequent condition. The study aimed to introduce a novel surgical technique and proposed our idea about enough surgical margin that could be more important for stage I lung cancer. Therefore, randomized controlled trials with large samples are necessary to gather accurate data and further confirm the feasible of the novel surgical technique and our ideas.

## Conclusion

Our results suggested that a two-port (mini-utility) VATS atypical segmentectomy (S^3^+S^1+2^c) with a tunneling stapler technique is an acceptable treatment option for patients with small-sized NSCLC located in left S^3^ close to the intersegmental plane between S^3^ and S^1+2^c. Accumulation of clinical VATS experience is also needed. Atypical segmentectomy should be determined by the target tumor location. Both preserving pulmonary function and obtaining adequate surgical margins should be taken into consideration.

## Data Availability Statement

The raw data supporting the conclusions of this article will be made available by the authors, without undue reservation.

## Ethics Statement

The studies involving human participants were reviewed and approved by the Ethics Committee of Shanghai Chest Hospital and Dehong People's Hospital. The patients/participants provided their written informed consent to participate in this study.

## Author Contributions

CZ and WL involved in conception and design of the research. CZ and JQ involved in acquisition, analysis, and interpretation of the data. CZ involved in statistical analysis. CZ, JQ, and WL involved in writing, review, and revision of the manuscript. All authors contributed to the article and approved the submitted version.

## Conflict of Interest

The authors declare that the research was conducted in the absence of any commercial or financial relationships that could be construed as a potential conflict of interest.

## Publisher's Note

All claims expressed in this article are solely those of the authors and do not necessarily represent those of their affiliated organizations, or those of the publisher, the editors and the reviewers. Any product that may be evaluated in this article, or claim that may be made by its manufacturer, is not guaranteed or endorsed by the publisher.
